# Supplementary material for: Toward asleep DBS: cortico-basal ganglia spectral and coherence activity during interleaved propofol/ketamine sedation mimics NREM/REM sleep activity
Source: NPJ Parkinsons Dis. 2021 Aug 2;7:67. doi: 10.1038/s41531-021-00211-9 (PMC8329235; doi:10.1038/s41531-021-00211-9)
Supplement: Supplementary file 2 — Reporting Summary [file 41531_2021_211_MOESM2_ESM.pdf]

nature research

Corresponding author(s): Jing Guang

Last updated by author(s): Jun 22, 2021

Reporting Summary

Nature Research wishes to improve the reproducibility of the work that we publish. This form provides structure for consistency and transparency in reporting. For further information on Nature Research policies, see our Editorial Policies and the Editorial Policy Checklist.

Statistics

For all statistical analyses, confirm that the following items are present in the figure legend, table legend, main text, or Methods section.

n/a	Confirmed	
☐	☒	The exact sample size (*n*) for each experimental group/condition, given as a discrete number and unit of measurement
☐	☒	A statement on whether measurements were taken from distinct samples or whether the same sample was measured repeatedly
☐	☒	The statistical test(s) used AND whether they are one- or two-sided<br>*Only common tests should be described solely by name; describe more complex techniques in the Methods section.*
☐	☒	A description of all covariates tested
☐	☒	A description of any assumptions or corrections, such as tests of normality and adjustment for multiple comparisons
☐	☒	A full description of the statistical parameters including central tendency (e.g. means) or other basic estimates (e.g. regression coefficient) AND variation (e.g. standard deviation) or associated estimates of uncertainty (e.g. confidence intervals)
☐	☒	For null hypothesis testing, the test statistic (e.g. *F*, *t*, *r*) with confidence intervals, effect sizes, degrees of freedom and *P* value noted<br>*Give P values as exact values whenever suitable.*
☒	☐	For Bayesian analysis, information on the choice of priors and Markov chain Monte Carlo settings
☒	☐	For hierarchical and complex designs, identification of the appropriate level for tests and full reporting of outcomes
☒	☐	Estimates of effect sizes (e.g. Cohen's *d*, Pearson's *r*), indicating how they were calculated

Our web collection on statistics for biologists contains articles on many of the points above.

Software and code

Policy information about availability of computer code

Data collection	The electrooculography, electromyography, electroencephalogram, local field potential and spiking activity in this study was collected by SnR software version, 2.0.0, Alpha Omega Engineering.<br>Live video used in this study was collected by ImagingSource, NC, USA and synchronized with electrophysiological data by SnR software version, 2.0.0, Alpha Omega Engineering.
Data analysis	All data analysis were performed by custom scripts on Matlab (MathWorks, Natick, MA; version R2018b). The custom scripts used in this study are available from corresponding author upon request.

For manuscripts utilizing custom algorithms or software that are central to the research but not yet described in published literature, software must be made available to editors and reviewers. We strongly encourage code deposition in a community repository (e.g. GitHub). See the Nature Research guidelines for submitting code & software for further information.

Data

Policy information about availability of data

All manuscripts must include a data availability statement. This statement should provide the following information, where applicable:

- Accession codes, unique identifiers, or web links for publicly available datasets
- A list of figures that have associated raw data
- A description of any restrictions on data availability

The data used in this study are available from corresponding author upon reasonable request.

Field-specific reporting

Please select the one below that is the best fit for your research. If you are not sure, read the appropriate sections before making your selection.

☒ Life sciences ☐ Behavioural & social sciences ☐ Ecological, evolutionary & environmental sciences

For a reference copy of the document with all sections, see nature.com/documents/nr-reporting-summary-flat.pdf

Life sciences study design

All studies must disclose on these points even when the disclosure is negative.

Sample size	Based on conventions of non-human primate researches, two monkeys were used in this study. For each monkey, multiple types of signals were recorded from several locations during several sedation sessions.
Data exclusions	The nights which EMG has no clear separation between NREM and REM were excluded. For spectrogram and coherogram, the data were excluded when relevant electrode was moving or defined as outliers (see method).
Replication	Two monkeys were used in this study, and results from each of them were qualitatively similar.
Randomization	Randomization was not relevant to this study, as there was no different experimental groups/conditions.
Blinding	Blinding was not relevant to this study, as there was no different experimental groups/conditions.

Reporting for specific materials, systems and methods

We require information from authors about some types of materials, experimental systems and methods used in many studies. Here, indicate whether each material, system or method listed is relevant to your study. If you are not sure if a list item applies to your research, read the appropriate section before selecting a response.

Materials & experimental systems

n/a	Involved in the study	
☒	☐	Antibodies
☒	☐	Eukaryotic cell lines
☒	☐	Palaeontology and archaeology
☐	☒	Animals and other organisms
☒	☐	Human research participants
☒	☐	Clinical data
☒	☐	Dual use research of concern

Methods

n/a	Involved in the study	
☒	☐	ChIP-seq
☒	☐	Flow cytometry
☒	☐	MRI-based neuroimaging

Animals and other organisms

Policy information about studies involving animals; ARRIVE guidelines recommended for reporting animal research

Laboratory animals	Two female African green monkeys (Chlorocebus aethiops sabaeus, Verets). Age, Ch, 7-8; Cs, 10-11.
Wild animals	No wild animals were used in this study.
Field-collected samples	No samples collected from the field in this study.
Ethics oversight	This study was approved and supervised by the Institutional Animal Care and Use Committee of the faculty of medicine, The Hebrew University of Jerusalem, Israel.

Note that full information on the approval of the study protocol must also be provided in the manuscript.
